# Phosphomannose isomerase affects the key enzymes of glycolysis and sucrose metabolism in transgenic sugarcane overexpressing the *manA* gene

**DOI:** 10.1007/s11032-015-0295-4

**Published:** 2015-03-15

**Authors:** Muqing Zhang, Xiaolei Zhuo, Jihua Wang, Chuanyu Yang, Charles A. Powell, Rukai Chen

**Affiliations:** 1 State Key Laboratory of Conservation and Utilization of Subtropical Agri-Biological Resources, Guangxi University, Nanning, 530005 China; 2 Fujian Agricultural and Forestry University, Fuzhou, 350002 China; 3 IRREC-IFAS, University of Florida, Fort Pierce, FL 34945 USA

**Keywords:** Sugarcane (*Saccharum officinarum* L.), Phosphomannose isomerase (PMI), Glycolysis, Sucrose accumulation/degradation, Transgenic plant

## Abstract

Sugarcane is one of the most important crops cultivated for the production of sugar and ethanol. In our previous studies, an innovative positive selection system for obtaining transgenic sugarcane, which utilized the *E. coli*-derived *manA* gene as the selectable marker and mannose as the selective agent, was developed and patented in China. In this paper, the influence of phosphomannose isomerase (PMI) overexpression on the key enzymes of both glycolysis and sucrose metabolism was investigated in transgenic sugarcane through the *manA* gene. Overexpressed PMI increased hexokinase activity by approximately 24 % compared with non-transgenic control plants, but pyruvate kinase (PK) activity was reduced by approximately 14 %. In comparison with the non-transgenic control plants, the activities of sucrose synthase, sucrose-phosphate synthase, and acid invertase were also modestly affected in the PMI-overexpressing transgenic plants, but no significant differences were observed at the stalk elongation and maturity stages. However, agronomic and technical traits were not affected by *manA* gene overexpression in the transgenic sugarcane. In conclusion, PMI overexpression significantly affected the hexokinase and PK activities by catalyzing the reversible interconversion between mannose-6-phosphate and fructose-6-phosphate, which is an intermediate of glycolysis. However, it had no significant effects on sucrose accumulation in sugarcane.

**Electronic supplementary material:**

The online version of this article (doi:10.1007/s11032-015-0295-4) contains supplementary material, which is available to authorized users.

## Introduction

Sugarcane is a major crop for the production of sucrose and ethanol in the tropical and subtropical regions of more than 100 countries. In 2012/2013, 14.6 million metric tons (MMT) of sugar was produced in China and more than 90 % was derived from sugarcane (Sugar: World Markets and Trade, http://usda01.library.cornell.edu/usda/fas/sugar//2010s/2012/sugar-11-20-2012.pdf). The genetic complexity and low fertility of sugarcane render conventional breeding methods laborious, and therefore, sugarcane is a prime candidate for improvements through genetic engineering (Singh et al. 2013; Ming et al. 2006). Genetic transformation offers the potential to introduce elite commercial sugarcane varieties with new and desirable characteristics, such as resistance to pathogens and abiotic stress, as well as improvements in their agronomic performance and sugar yield (Groenewald and Botha 2008; Chong et al. 2007; Zhang et al. 2006). Herbicide- and virus-resistant transgenic sugarcane plants have been tested in the field, but no commercial release of these plants has occurred to date (Ruan et al. 2007; Singh et al. 2013).

The use of *manA*/mannose as a positive selection system for plant transformation has been reported in many plant species (Lamblin et al. 2007; Jain et al. 2007). The *manA* gene encodes phosphomannose isomerase (PMI), which interconverts mannose-6-phosphate to fructose-6-phosphate, thus enabling the transformed plant cells to assimilate mannose via glycolysis (Thiruvengadam et al. 2011). The gene transfer and transgenic method based on the *manA* gene have been patented for sugarcane transformation in China (Zhang et al. 2007). Although Privalle (2002) and Reed et al. (2001) summarized the biochemistry, mode of action, and safety issues associated with PMI, there have been no reports on the influence of *manA* overexpression on sucrose synthesis/degradation or glycolysis.

Sugarcane stores high concentrations of sucrose in the internodes via sucrose translocation and accumulation (Mao et al. 2006). Acid invertase (AI), neutral invertase (NI), sucrose synthase (SuSy), and sucrose-phosphate synthase (SPS) are key enzymatic regulators for sugar accumulation and degradation in storage parenchyma of sugarcane stalk (Bosch et al. 2004). Therefore, this study focused on glycolysis and sucrose metabolism in transgenic sugarcane mediated by the *manA* gene. PMI transgenic sugarcane was used to explore the effects of *manA* overexpression on several key enzymes in sugar metabolism throughout plant growth and development.

## Materials and methods

### Plant materials

The sugarcane plants used in these experiments were transgenic lines (tp7, tp13, and tp29) and non-transgenic plants from the seed-cane (wt) and tissue-cultured (tc) plants of Funong 95-1702 (*Saccharum* spp. hybrids).

1. Three independent transgenic lines tagged “tp7, tp13, and tp29” were *manA*-overexpression lines generated by particle bombardment-mediated transformation with a *ubi*-*1* promoter-trap vector. The *manA* expression in these lines was confirmed and quantified using a chlorophenol red assay, enzymatic analysis, and quantitative RT-PCR detection. The plasmid pUBI-PMI, *manA* (or *pmi*) gene transfer, and transgenic protocol were described in our previous study (Zhang et al. 2007, 2014).
2. Non-transgenic lines (control plants) were generated from seed-cane (wt, receptor of gene transfer) and tissue-cultured plants (tc) of Funong 95-1702.

All of the above transgenic lines (tp7, tp13, and tp29) and non-transgenic control plants (wt and tc) were grown in the fields of Fujian Agriculture and Forestry University in Fuzhou, China, using a randomized complete block design with three replicates. Each replicate consisted of one plot per line with 4 rows that were 6 m long and spaced 1.1 m apart.

### Polymerase chain reaction (PCR) amplification of manA transgenic sugarcane lines

Total RNA was extracted using the TRIzol^®^ Plus RNA Purification Kit (Gibco BRL, Grand Island, NY, USA) according to the manufacturer’s protocol. RNA was treated with DNase (Takara, Bio. Inc., Dalian, China) to eliminate DNA contamination, adjusted to uniform concentrations, and then reverse-transcribed with avian myeloblastosis virus (AMV) reverse transcriptase (Takara, Bio. Inc., Dalian, China). The synthesized first-strand cDNA was used to amplify a *manA* gene fragment from the transgenic plants. The RT-PCR product was purified using a TaKaRa PCR Fragment Recovery Kit (Takara, Bio. Inc., Dalian, China) and cloned into the compatible sites of pMD-18T (Takara, Bio. Inc., Dalian, China) using T_4_ DNA ligase (Takara, Bio. Inc., Dalian, China). After the product was transformed into competent *E. coli DH5α*, a single bacterial colony was chosen and cultivated in 3 mL LB broth with 100 mg L^−1^ of ampicillin and shaken at 250 rpm and 37 °C overnight. Recombinant plasmid was extracted using a TaKaRa MiniBest DNA Purification Kit (Takara, Bio. Inc., Dalian, China) according to the manufacturer’s instructions and identified by PCR and restriction endonuclease enzyme digestion. The inserted *manA* gene from the recombinant plasmid was then sequenced by Takara (Takara, Bio. Inc., Dalian, China), and the recovered sequences of the *manA* gene and their corresponding amino acid sequences were blasted against the GenBank database.

Transcripts of the *manA* gene were quantified by qRT-PCR using cDNA synthesized with ExScript™ RTase (SYBR ExScript RT-PCR Kit, Takara, Bio Inc., Dalian, China). The following primer pairs were used for PCR:

For the target *manA* gene (*pmi*-*f* 5′-ACT,AAG,GTC,ATG,CAG,CGA,AGG and *pmi*-*r* 5′-GGA,ACT,GGT,TCC,AAT,GTG,AAA); for the internal control 25s rRNA gene (*25s rRNA*-*f* 5′-GCA,GCC,AAG,CGT,TCAT,AGC and *25s rRNA*-*r* 5′-CCT,ATT,GGT,GGG,TGA,ACA,ATC,C).

Quantitative PCRs were performed in a total volume of 25 μL containing 12.5 µl of SYBR Primix ExTaq™ (2×), 500 nM each primer (0.5 µl each), 2.5 µl of cDNA template, and 9 µl of sterilized ddH_2_O in a MiniOpticon™ instrument (Bio-Rad, CA, USA) using the following cycling program: 95 °C for 10 min, followed by 40 cycles of 94 °C for 5 s and 60 °C for 25 s. Data were acquired every 0.2 °C at the interval from 55 to 90 °C. Three replicates were performed on each calibrator or sample and on no-template negative controls. *C*_*t*_ values were generated for amplification curves of both the target *manA* gene and *25s rRNA* internal control gene in each sample using the MiniOpticon™ data analysis package in the second derivative maximum mode of analysis. The averaged *C*_*t*_ values of three replicates in each run were determined from the calibrator sample for both the target *manA* gene and the internal control *25s rRNA* gene. Fold changes (relative expression) for the *manA* relative to the calibrator sample (control plant) were then calculated according to the Livak method (Livak and Schmittgen 2001).

### Measurement of PMI activity

Specific PMI activity was quantified using the procedure described by Wang et al. (2000). Briefly, fresh leaf tissue (500 mg) was pulverized and extracted in 1.5 mL of ice-cold 50 mM Tris–HCl (pH 7.5) containing 1 mM phenylmethylsulfonyl fluoride (PMSF) and 1 mM polyvinylpyrrolidone (PVP). The extract was centrifuged at 15,000×*g* for 20 min at 4 °C. To start the reaction, 100 μL of the supernatant was added to a mixture of 65 μL of 50 mM Tris–HCl (pH 7.5), 50 μL of 10 mM nicotinamideadenine dinucleotide phosphate (NADP), 50 μL of 10 U glucose-6-phosphate isomerase (GPI, EC5.3.1.9, Sigma, St. Louis, MO, USA), 25 μL of 10 U glucose-6-P dehydrogenase (G6PD, EC1.1.1.49, Sigma, St. Louis, MO, USA), and 10 μL of 50 mM d-mannose-6-P. The reaction mix was then measured at 340 nm in a spectrophotometer (Metrospec, V. A. Howe Ltd., London). A blank control was used in which the d-mannose-6-P was omitted. One unit of PMI activity, defined as micromoles of substrate turnover per minute, was calculated using the extinction coefficient for NADPH (6.22 mM^−1^ cm^−1^).

Chlorophenol red (CPR) assays were performed to qualitatively verify PMI activity (Wright et al. 2001). Sections of the shoot tips (0.5 cm in length), leaves (0.5 cm^2^ in area), and roots (0.5 cm in length) from the transgenic (tp-29, tp-13, and tp-7) as well as non-transgenic from tissue-cultured (tc) and wild-type (wt) plants were surface-sterilized with 70 % ethanol (v/v) for approximately 3 min and with 20 % (v/v) sodium hypochlorite for 15 min. The samples were then rinsed with distilled water three times, transferred to MS medium (pH 6.4) with 5 g L^−1^ mannose and 50 mg L^−1^ CPR (Chlorophenol Red, Sigma, St. Louis, MO, USA) dye, and incubated at 29 °C in the dark for 2 days. Acidification of the medium by mannose-resistant tissue resulted in a color change of the medium from purple (or red) to yellow (or orange) in the transgenic samples.

### Activity assays of glycolytic and sucrose metabolism enzymes

Fully expanded young leaves were sampled from the field-grown transgenic line (tp-7) and the non-transgenic control plants (tc and wt) at the stages of tillering, elongation, and maturation. Frozen leaf tissues were weighed and ground to a fine powder with liquid nitrogen in a chilled mortar. Extraction buffer was then added to the ground sample, and the slurry was ground again in order to thoroughly mix the powdered tissue and the buffer. The extraction buffer used for measuring the activities of hexokinase (HXK, E.C. 2.7.1.1) and pyruvate kinase (PK, EC 2.7.1.40) contained 50 mM HEPES (pH 7.5), 12 mM MgCl_2_, 1 mM EDTA, 1 mM EGTA, 10 mM DTT, 2 mM benzamidine, 2 mM *N*-aminocapronate, and 10 mM diethyldithiocarbamate. The homogenate was centrifuged at 15,000×*g* for 10 min at 4 °C, and the supernatant was immediately desalted on a Sephadex G-25 column (Pharmacia PD-10, Pittsburgh, PA, USA). Diluted extracts were concentrated with a Centricon-30 (Millipore Corporation, Billerica, MA, USA) at 4 °C, and the assay was then immediately performed (Goldner et al. 1991). HXK activity was assayed at 340 nm by adding a solution containing 5 mM ATP, 0.5 mM NADP, 7.5 mM MgSO_4_, 100 mM KCl, 5 mM β-mercaptoethanol, and 2.5 mM glucose. The enzyme reaction was initiated by adding 0.2 U of G6PD (EC1.1.1.49, Sigma, St. Louis, MO, USA). PK activity was measured at 340 nm in a solution of 25 mM Mes-HCl (pH 6.5), 2 mM PEP, 1 mM ADP, 20 mM KCl, 10 mM MgCl_2_, 0.15 mM NADH, 1 mM DTT, and 5 % PEG-8000. The PK enzyme reaction was initiated by the addition of 2U of lactate dehydrogenase (LDH; EC 1.1.1.27, Sigma, St. Louis, MO, USA). The tissue extractions and assays for sucrose metabolism enzymes were performed using the methods described by Gutierrez-Miceli et al. (2002). Activity of sucrose-phosphate synthase (SPS: E.C. 2.4.1.14) was measured in a reaction mixture containing 100 mM HEPES–NaOH (pH 7.5), 5 mM MgCl_2_, 1 mM EDTA, 4 mM fructose-6-P (F6P), 20 mM glucose-6-P (G6P), and 3 mM UDP-glucose (UDPG) (Kohler et al. 1988). The mixture was incubated at 30 °C for 30 min, and then the reaction was terminated by adding 70 μL of 30 % (w/v) KOH. Sucrose formation was estimated as described by van Handel (1968). Sucrose synthase (SuSy: E.C. 2.4.1.13) activity was also measured with different reaction mixtures including 100 mM HEPES–NaOH (pH 7.5), 5 mM MgCl_2_, 1 mM EDTA, 4 mM fructose, and 3 mM UDPG. Activity of acid invertase (AI: E.C. 3.2.1.26) was measured at 37 °C upon the addition of 0.5 mL of desalted extract to 0.5 mL of 1 M sodium acetate (pH 4.5). The enzyme reaction was initiated by the addition of 1 mL of 120 mM sucrose solution. The mixture was then incubated at 30 °C, and the amount of reducing sugars formed was measured using the dinitrosalicylic acid (DNSA) method after 1 h of incubation (Hatch et al. 1963). The total protein content of the dialyzed extracts was determined using the method described by Bradford using bovine serum albumin as a standard (Bradford 1976).

### Agronomic and technical traits in the transgenic sugarcane

One month before harvest, 30 stalks were randomly chosen for length and diameter measurements. Ten stalks of each sample were then processed through a three-roller mill under a pressure of 500 kg cm^−3^. A portion of the crusher juice was analyzed for Brix (percent soluble solids, w/w) with a refractometer. Polarization of the clarified juice was tested with an automated saccharimeter (SUCROMAT^*®*^ Digital Automatic Saccharimeter, GRAZ, Österreich). Brix and polarization values were used to estimate sucrose content.

### Statistics analysis

Variance analysis was conducted to assess variation among transgenic (tp 7) and non-transgenic control plants (tc and wt) using the SAS/STAT procedure ANOVA. Differences among transgenic (tp 7) and non-transgenic control plants (tc and wt) at each growth stage were assessed by Duncan’s multiple range test at *Pr* ≤ 0.05. Data presented here are the averaged values from three replicates.

## Results

### Overexpression of manA in sugarcane transgenic lines

**Fig. 1:**
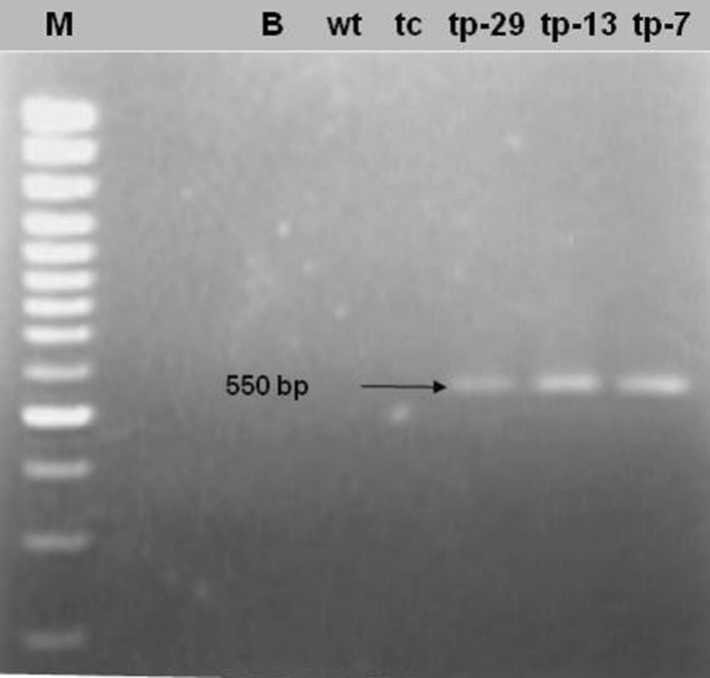
RT-PCR amplification of the *manA* gene from three transgenic lines and non-transgenic control plants. *Arrow* shows the amplified 550 bp *manA* fragment. *Lanes* *M*: GeneRuler 1-kb DNA Ladder (SM0311); *B*: blank control (water); non-transgenic control plants from seed-cane (*wt*) and tissue-cultured (*tc*) plants; transgenic lines (*tp*-*29*, *tp*-*13,* and *tp*-*7*)

The expected *manA* RT-PCR product of 550 bp was observed in cDNA amplified from leaves of the transgenic lines (tp-29, tp-13, and tp-7). No bands were detected in the non-transgenic plants (wt and tc) (Fig. 1). The RT-PCR products sequenced and subsequently blasted against GenBank showed 100 % identity with the nucleic acid sequence of the *manA* gene, confirming that the *manA* gene was integrated and expressed in the three transgenic sugarcane lines (tp-29, tp13, and tp-7).

**Fig. 2:**
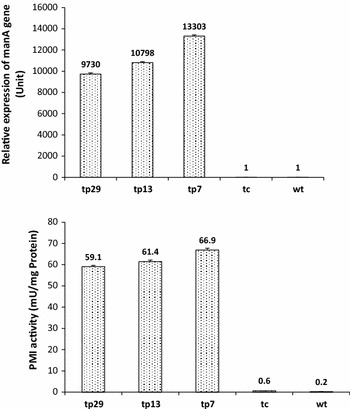
Quantitative real-time RT-PCR detection of *manA* gene expression and specific enzyme activity of phosphomannose isomerase (PMI) in the transgenic lines (tp-29, tp-13, and tp-7) and non-transgenic control plants from seed-cane (wt) and tissue-cultured (tc) plants. The *manA* gene was considered differentially expressed in this article if its relative expression was twofold or greater. No enzyme activity was detected in the non-transgenic control plants from seed-cane (wt) and tissue-cultured (tc) plants

The relative expression (fold change) of *manA* in the transgenic lines ranged from 9,730 (tp-13) to 13,303 (tp-7) and correlated significantly with PMI activity (*r* = 0.9999, *Pr* ≤ 0.0001) (Fig. 2). The PMI activity in the transgenic lines at tillering stages was 57.90 mU mg^−1^ for tp-7, 49.60 mU mg^−1^ for tp-13, and 53.70 mU mg^−1^ for tp-29. No PMI enzyme activity was quantified in the non-transgenic plants (wt and tc), indicating that PMI was absent in the non-transgenic sugarcane (Fig. 2).

**Fig. 3:**
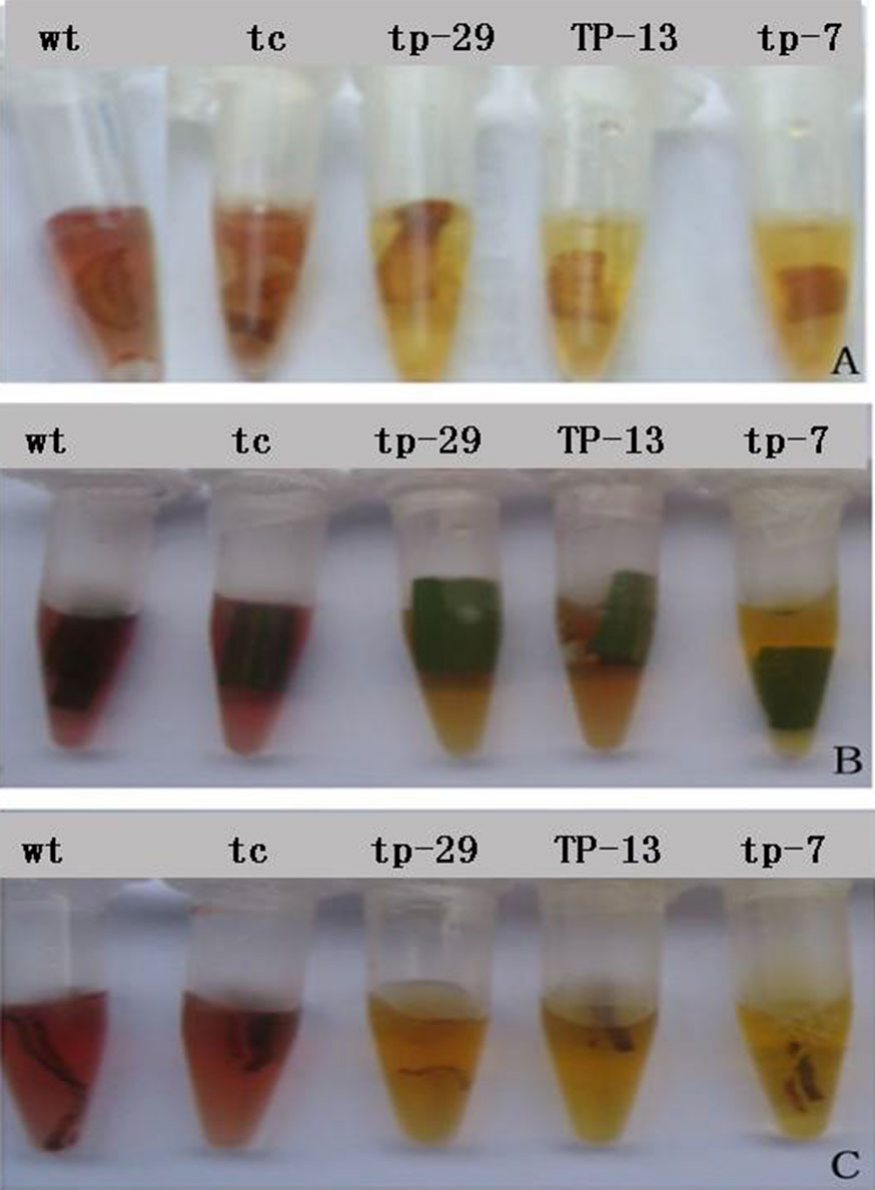
Chlorophenol red dye assay for PMI activity in shoot tip (**a**), young leaf (**b**), and root (**c**) sections of sugarcane. Non-transgenic control plants from seed-cane (wt) and tissue-cultured (tc) plants; transgenic lines (tp-29, tp-13, and tp-7). *Red*/*purple* color in medium indicates little or no PMI activity; *yellow*/*orange* in medium indicates enzyme activity. (Color figure online)

Using a chlorophenol red assay, gradual color changes from purple to yellow or orange were observed only in media containing sections of leaf, shoot tip, and root from all transgenic lines (tp-29, tp-13, and tp-7) (Fig. 3). However, no color change was observed in samples derived from the non-transgenic control plants (wt and tc), indicating that the non-transgenic control plants (wt or tc) could not metabolize the mannose.

### Glycolysis and sucrose synthesis/degradation in the PMI-overexpressing transgenic sugarcane

**Fig. 4:**
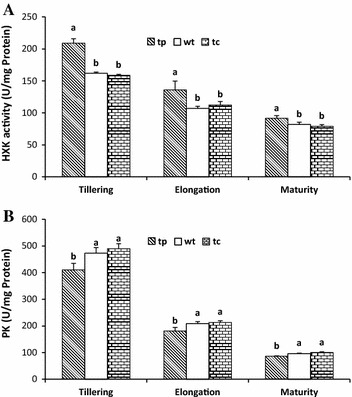
Specific activities of hexokinase (HXK) (A) and pyruvate kinase (PK) (B) in the leaves of transgenic plants (tp-7) overexpressing *manA* gene, and non-transgenic control plants from seed-cane (wt) and tissue-cultured (tc) plants at different stages of growth and development. The different lowercase letters indicate significant differences at the level of *Pr* ≤ 0.05

**Fig. 5:**
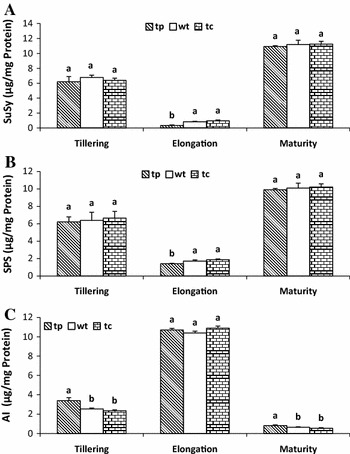
Specific activities of sucrose synthase (SuSy) (**a**), sucrose-phosphate synthase (SPS) (**b**), and acid invertase (AI) (**c**) in the leaves of transgenic plants (tp-7) overexpressing *manA* gene, and non-transgenic control plants from seed-cane (wt) and tissue-cultured (tc) plants at different stages of growth and development. Different *lowercase letters* indicate significant differences at the level of *Pr* ≤ 0.05

Variation in the specific activities of carbohydrate metabolism enzymes between the transgenic line (tp-7) and non-transgenic control plants (wt and tc) reflected the influence of *manA* overexpression on glycolysis and sucrose synthesis/degradation (Figs. 4, 5). Hexokinase (HXK) activity in the transgenic line was significantly greater than that in the non-transgenic control plants (wt and tc), and there was no difference between the seed-cane plant (wt) and the tissue-cultured plants (tc) (Fig. 4). HXK activity in both transgenic and non-transgenic plants diminished significantly during development, with an approximately twofold lower level at maturity compared with that at the tillering and elongating stages. Similar to HXK, pyruvate kinase (PK) activity also decreased throughout development, with a fourfold lower level at maturity compared with that at the tillering stage.

Activity of acid invertase (AI) was highest at the elongating stage followed by the tillering stage and was lowest at maturity. Compared with the non-transgenic control plants (wt and tc), the transgenic line with the *manA* gene had no significant difference in AI activity at the elongating stage, but a significant difference at the tillering and maturity stages (Fig. 5c). This trend was inversely related to the activity of sucrose synthase (SuSy) and sucrose-phosphate synthase (SPS) (Fig. 5a, b), as higher AI activity correlated with lower SuSy and SPS activity over sugarcane growth and development. No significant differences in SuSy and SPS activities between transgenic and non-transgenic plants were found at the tillering and maturity stages, but a significant difference was found at the elongating stage (*Pr* ≤ 0.05). The activity of both SPS and SuSy was greatest during the maximum accumulation of sucrose, which occurred at the maturing stage, while AI activity was almost undetectable. The maximum observed activity of SPS and SuSy occurred at maturity, and the highest activity of AI occurred at the elongating stage. No significant differences in sucrose content, brix, plant height, or stalk diameter were observed between transgenic lines and non-transgenic control plants (Table 1). This suggested that overexpression of the *manA* gene had no significant influence on sugarcane growth and sucrose storage.

**Table 1:** Agronomic and technical traits of transgenic and non-transgenic sugarcane plants 1 month before harvest

Characteristics	Transgenic line (tp7)	Non-transgenic plants	
		Seed-cane plant (wt)	Tissue-cultured plant (tc)
Stalk diameter (cm)	2.86 ± 0.27a^ζ^	2.91 ± 0.19a	2.75 ± 0.38a
Plant height (cm)	312 ± 5.89a	309 ± 10.54a	293 ± 10.25a
Brix (%)	21.37 ± 1.05a	21.38 ± 0.87a	21.05 ± 1.23a
Gravity purity (%)	94.75 ± 0.12a	94.21 ± 0.05a	93.67 ± 0.17a
Sucrose content (%)	16.67 ± 0.04a	16.93 ± 0.07a	16.48 ± 0.09a

Data are presented as mean ± standard error

^ζ^Genotypes with same letters within traits are not significantly different as judged by Duncan’s multiple range test at *Pr* ≥ 0.05

## Discussion and conclusion

Selectable marker genes have been pivotal in the development of plant transformation technologies because the marker genes allow scientists to identify or isolate cells that are expressing the cloned DNA (Wilmink and Dons 1993). For sugarcane gene transfer and transgenic lines, the most frequently used selectable marker genes are antibiotic resistance genes (*nptII* or *hpt*) (Bower et al. 1996), herbicide resistance genes (*bar*) (Falco et al. 2000), or the phosphomannose isomerase gene (*manA*) (Jain et al. 2007; Zhang et al. 2014).

PMI has utility as a selectable marker for transformation of many plant species (Joersbo et al. 1998) because expression of the *E. coli manA* gene encoding PMI allows cells to utilize mannose as a carbon source and survives on media containing mannose. PMI converts mannose-6-phosphate to fructose-6-phosphate so that mannose can be efficiently taken up and phosphorylated to mannose-6-phosphate by HXK (Miles and Guest 1984), which is associated with glycolysis and sucrose metabolism (Fig. 5). Sugarcane cannot metabolize mannose in nature because PMI is absent in sugarcane (Jain et al. 2007; Zhang et al. 2014). Sugarcane stores fixed carbon as soluble sugars, mostly sucrose, in vacuoles of parenchyma cells of mature stalks (Rae et al. 2009). Transforming photosynthetically fixed carbon into stored sucrose is a complex process that involves osmotic regulation, sugar sensors, sucrose cleavage, and feedback regulation of photosynthesis (McCormick et al. 2008). Therefore, the effects on sucrose storage or glycolysis would be addressed through the application of *manA* in sugarcane transformation in the sugarcane industry.

The data presented here indicate that the overexpression of *manA* driven by the *ubi*-*1* promoter in transgenic sugarcane (tp7, tp13, and tp29) significantly increased PMI activity, resulting in an interconversion between fructose-6-phosphate and mannose-6-phosphate. Compared with the non-transgenic control plants, the transgenic sugarcane lines possessing the *manA* gene (tp7) had significantly greater HXK activity, but less PK activity, whereas there was no difference in the non-transgenic control plants (wt and tc) (Fig. 4). HXK activity in both transgenic and non-transgenic plants diminished significantly during development, with an approximately twofold lower level at maturity compared with the tillering and elongating stages. Similar to HXK, PK activity also decreased throughout development, with a fourfold lower level at maturity compared with the tillering stage. HXK (EC2.7.1.1) catalyzes the phosphorylation of hexoses (such as glucose, fructose, and mannose) to hexose monophosphates, which are the substrates for a limited number of intracellular metabolic processes, such as glycolysis. Glucose can be readily metabolized beyond the HXK step (Karve et al. 2010), and the resulting G6P is able to cross the cell membrane due to the addition of a charged phosphate group. However, mannose can also be phosphorylated by HXK, and the resulting phosphomannose pool is relatively inert. Free mannose is generally absent in most green plants during development, except for the breakdown of reserve mannose in seeds or other storage organs (Ciereszko and Kleczkowski 2002; Herold and Lewis 1977). Therefore, glucose and fructose are the major substrates for hexokinase. The transfer of a phosphate group from phosphoenolpyruvate (PEP) to ADP is catalyzed by PK, yielding one pyruvate and one ATP in the final step of glycolysis (Supplementary Fig. S1) (Meza et al. 2012). The absence of PK slows down the process of glycolysis (Rose 1970). This indicates that more sucrose is degraded via glycolysis to produce energy for sugarcane growth at early stages. In contrast to HXK, PK activity decreased by approximately 13 % in the transgenic line during all of the development stages (Fig. 4). This decrease in PK activity should produce fewer pyruvate molecules, resulting in reduced ATP formation via the Krebs cycle and the electron transport system. This result suggests that products of glycolysis might be reduced in PMI-overexpressing transgenic plants, and more mannose-6-phosphate is likely produced for glycosylation via phosphomannomutase.

Sucrose is the major product of sugarcane. High concentrations of sucrose are stored in the internodes of sugarcane via sucrose translocation and accumulation. Sucrose metabolism is regulated by several key enzymes, such as invertase, SuSy, and SPS (Chandra et al. 2012) (Bosch et al. 2004; Mao et al. 2006) (Supplementary Fig. S1). The results presented here showed that enzyme activities of SPS and SuSy were greatest during the maturing stage when the largest accumulation of sucrose took place, whereas AI activity was at its lowest at this stage (Fig. 5). However, the abundant activities of SPS and SuSy at the maturing stage and of AI at the elongating stage were not significantly different between transgenic line (tp7) and non-transgenic control plants, indicating that sucrose accumulation at maturity and sucrose degradation at elongation were not markedly affected by overexpression of the manA gene in transgenic plants. Invertases, which cleave sucrose to glucose and fructose, are classified as acid or neutral invertase (AI or NI) on the basis of their optimum pH. Our results indicated that AI activity was highest during the elongating stage, followed by the early tillering stage, and lowest during the maturing stage (Fig. 5). Variation in AI activity during sugarcane development is thought to function as a significant regulatory mechanism of sucrose breakdown. During stem elongation when sugarcane grows fast, the plant needs more carbon sources for biochemical activities and, correspondingly, AI activity is higher and sucrose concentration is lower. Transformation with the sugarcane antisense *sc*-*invh* gene produced a cell line with moderately inhibited AI activity and a twofold increase in sucrose accumulation (Sugiharto et al. 1997). Sucrose accumulation in the stalk was regulated by down-regulation of AI and up-regulation of SPS (Chandra et al. 2012; Zhu et al. 1997). SPS is believed to be a key enzyme controlling the biosynthesis of sucrose in higher plants by catalyzing the formation of sucrose-6-phosphate from fructose-6-phosphate and UDP-glucose (Huber and Huber 1996). During the elongating stage, the activities of both SPS and SuSy in the transgenic line (tp7) were significantly lower than those in the non-transgenic plants, indicating that less sucrose was accumulated at the elongation stage of sugarcane in the transgenic line. However, both SPS and SuSy activities were not significantly different between transgenic line and non-transgenic controls. The results in Table 1 also showed that no marked influence of *manA* gene on sugarcane growth and sucrose storage was detected between the transgenic plants and the non-transgenic control plants, such as sucrose content, brix, plant height, and stalk diameter. Compared with their conventional counterparts, no adverse effects on growth or other agronomic characteristics were found in PMI transgenic corn and wheat (Wright et al. 2001; Negrotto et al. 2000; Wang et al. 2000), sugar beet (Joersbo et al. 1998), and rice (Lucca et al. 2001; He et al. 2006).

In conclusion, *manA*/mannose as a positive selection system involving intermediary metabolism is a better system for plant transformation than traditional antibiotic or herbicide selection systems. No obvious qualitative differences in growth or other agronomic and technical traits were observed in transgenic sugarcane compared with the non-transgenic control plants. Compared with the non-transgenic control plants, PMI-overexpressed transgenic sugarcane significantly increased HXK activity by 10–25 % at all growth stages and AI by 20–35 % at the tillering and maturity stages, but decreased PK activity by 10–20 % throughout development and SuSy by over 140 % and SPS by 20–30 % during the elongation stages. Although the activities of SuSy and SPS were affected to a small extent in the PMI-overexpressing transgenic plants, no significant differences were observed in stalk elongation and sucrose accumulation. However, the key enzymes associated with glycolysis were significantly affected in transgenic sugarcane possessing the *manA* gene.

## Electronic supplementary material

Below is the link to the electronic supplementary material.

Supplementary material 1 (DOCX 127 kb)

## References

[CR1] Bosch S, Grof C, Botha F (2004). Expression of neutral invertase in sugarcane. Plant Sci.

[CR2] Bower R, Elliott AR, Potier BA, Birch RG (1996). High-efficiency, microprojectile-mediated cotransformation of sugarcane, using visible or selectable markers. Mol Breed.

[CR3] Bradford MM (1976). A rapid and sensitive method for the quantitation of microgram quantities of protein utilizing the principle of protein-dye binding. Anal Biochem.

[CR4] Chandra A, Jain R, Solomon S (2012). Complexities of invertases controlling sucrose accumulation and retention in sugarcane. Curr Sci.

[CR5] Chong BF, Bonnett GD, Glassop D, O’Shea MG, Brumbley SM (2007). Growth and metabolism in sugarcane are altered by the creation of a new hexose phosphate sink. Plant Biotechnol J.

[CR6] Ciereszko I, Kleczkowski LA (2002). Glucose and mannose regulate the expression of a major sucrose synthase gene in Arabidopsis via hexokinase-dependent mechanisms. Plant Physiol Biochem.

[CR7] Falco M, Neto AT, Ulian E (2000). Transformation and expression of a gene for herbicide resistance in a Brazilian sugarcane. Plant Cell Rep.

[CR100] Goldner W, Thom M, Maretzki A (1991) Sucrose metabolism in sugarcane cell suspension cultures. Plant Sci 73:143–147

[CR8] Groenewald JH, Botha FC (2008). Down-regulation of pyrophosphate: fructose 6-phosphate 1-phosphotransferase (PFP) activity in sugarcane enhances sucrose accumulation in immature internodes. Transgenic Res.

[CR101] Gutierrez-Miceli FA, Rodriguez-Mendiola MA, Ochoa-Alejo N, Mendez-Salas R, Dendooven L, Arias-Castro C (2002) Relationship between sucrose accumulation and activities of sucrose phosphatase, sucrose synthase, neutral invertase, and soluble acid invertase in micropropagated sugarcane plants. Acta Physiol Plant 24:441–446

[CR9] Hatch M, Sacher J, Glasziou K (1963). Sugar accumulation cycle in sugar cane. I. Studies on enzymes of the cycle. Plant Physiol.

[CR10] He Z, Duan Z, Liang W, Chen F, Yao W, Liang H, Yue C, Sun Z, Chen F, Dai J (2006). Mannose selection system used for cucumber transformation. Plant Cell Rep.

[CR11] Herold A, Lewis D (1977). Mannose and green plants: occurrence, physiology and metabolism, and use as a tool to study the role of orthophosphate. New Phytol.

[CR12] Huber SC, Huber JL (1996). Role and regulation of sucrose-phosphate synthase in higher plants. Annu Rev Plant Biol.

[CR13] Jain MK, Chengalrayan A, Abouzid A, Gallo M (2007). Prospecting the utility of a PMI/mannose selection system for the recovery of transgenic sugarcane (*Saccharum* spp. hybrid) plants. Plant Cell Rep.

[CR14] Joersbo M, Donaldson I, Kreiberg J, Petersen SG, Brunstedt J, Okkels FT (1998). Analysis of mannose selection used for transformation of sugar beet. Mol Breed.

[CR15] Karve R, Lauria M, Virnig A, Xia X, Rauh BL, Moore BD (2010). Evolutionary lineages and functional diversification of plant hexokinases. Mol Plant.

[CR16] Kohler EK, Thom JM, Maretzki A (1988). Activity of sucrose-phosphate synthase in sugar cane leaves. Phytochemistry.

[CR17] Lamblin F, Aimé A, Hano C, Roussy I, Domon JM, Van Droogenbroeck B, Lainé E (2007). The use of the phosphomannose isomerase gene as alternative selectable marker for Agrobacterium-mediated transformation of flax (*Linum usitatissimum*). Plant Cell Rep.

[CR18] Livak KJ, Schmittgen TD (2001). Analysis of relative gene expression data using real-time quantitative PCR and the 2^−∆∆CT^ method. Methods.

[CR19] Lucca P, Ye X, Potrykus I (2001). Effective selection and regeneration of transgenic rice plants with mannose as selective agent. Mol Breed.

[CR20] Mao L, Que F, Wang G (2006). Sugar metabolism and involvement of enzymes in sugarcane (*Saccharum officinarum* L.) stems during storage. Food Chem.

[CR21] McCormick AJ, Cramer MD, Watt DA (2008). Regulation of photosynthesis by sugars in sugarcane leaves. J Plant Physiol.

[CR22] Meza E, Becker J, Bolivar F, Gosset G, Wittmann C (2012). Consequences of phosphoenolpyruvate: sugar phosphotransferase system and pyruvate kinase isozymes inactivation in central carbon metabolism flux distribution in *Escherichia coli*. Microb Cell Fact.

[CR23] Miles JS, Guest JR (1984). Nucleotide sequence and transcriptional start point of the phosphomannose isomerase gene (manA) of *Escherichia coli*. Gene.

[CR24] Ming R, Moore PH, Wu K, Hont AD, Glaszmann JC, Tew TL, Mirkov TE, Da Silva J, Jifon J, Rai M (2006). Sugarcane improvement through breeding and biotechnology. Plant Breed Rev.

[CR25] Negrotto D, Jolley M, Beer S, Wenck A, Hansen G (2000). The use of phosphomannose-isomerase as a selectable marker to recover transgenic maize plants (*Zea mays* L.) via Agrobacterium transformation. Plant Cell Rep.

[CR26] Privalle LS (2002). Phosphomannose isomerase, a novel plant selection system. Ann N Y Acad Sci.

[CR27] Rae AL, Jackson MA, Nguyen CH, Bonnett GD (2009). Functional specialization of vacuoles in sugarcane leaf and stem. Trop Plant Biol.

[CR28] Reed J, Privalle L, Powell ML, Meghji M, Dawson J, Dunder E, Sutthe J, Wenck A, Launis K, Kramer C (2001). Phosphomannose isomerase: an efficient selectable marker for plant transformation. In Vitro Cell Dev Biol Plant.

[CR102] Rose ZB (1970) Evidence for a phosphohistidine protein intermediate in the phosphoglycerate mutase reaction. Arch Biochem Biophys 140:508–513 10.1016/0003-9861(70)90095-0 5528743

[CR29] Ruan M, Xu Y, Zheng Y, Yang C, Zheng B, Guo Y, Chen R, Zhang M (2007). Effects on enzyme activities and microbe in rhizosphere soil of ScMV-CP transgenic sugarcane. Chin Agric Sci Bull.

[CR30] Singh RK, Kumar P, Tiwari NN, Rastogi J, Singh SP (2013). Current status of sugarcane transgenic: an overview. Adv Genet Eng.

[CR31] Sugiharto B, Sakakibara H, Sugiyama T (1997). Differential expression of two genes for sucrose-phosphate synthase in sugarcane: molecular cloning of the cDNAs and comparative analysis of gene ex/*789 ssion. Plant\\\ ++++-*/ Cell Physiol.

[CR32] Thiruvengadam M, Hsu WH, Yang CH (2011). Phosphomannose-isomerase as a selectable marker to recover transgenic orchid plants (Oncidium Gower Ramsey). Plant Cell Tissue Organ.

[CR33] Van Handel E (1968). Direct microdetermination of sucrose. Anal Biochem.

[CR34] Wang A, Evans R, Altendorf P, Hanten J, Doyle M, Rosichan J (2000). A mannose selection system for production of fertile transgenic maize plants from protoplasts. Plant Cell Rep.

[CR35] Wilmink A, Dons JJM (1993). Selective agents and marker genes for use in transformation of monocotyledonous plants. Plant Mol Biol Rep.

[CR36] Wright M, Dawson J, Dunder E, Suttie J, Reed J, Kramer C, Chang Y, Novitzky R, Wang H, Artim-Moore L (2001). Efficient biolistic transformation of maize (*Zea mays* L.) and wheat (*Triticum aestivum* L.) using the phosphomannose isomerase gene, PMI, as the selectable marker. Plant Cell Rep.

[CR37] Zhang MQ, Wang HZ, Bai C (2006). Genetic improvement and breeding technique for sugar crop.

[CR38] Zhang MQ, Wu Y, Chen RK, Zuo XL (2007) A method to rapidly breed the transgenic sugarcane mediated with PMI gene. China patent ZL200710008719.X

[CR39] Zhang MQ, Zhou XL, Wang JH, Wu Y, Yao W, Chen RK (2014). Effective selection and regeneration of transgenic sugarcane plants using positive selection system. In Vitro Cell Dev Biol Plant.

[CR40] Zhu YJ, Komor E, Moore PH (1997). Sucrose accumulation in the sugarcane stem is regulated by the difference between the activities of soluble acid invertase and sucrose phosphate synthase. Plant Physiol.

