# Supplementary material for: Phosphomannose isomerase affects the key enzymes of glycolysis and sucrose metabolism in transgenic sugarcane overexpressing the manA gene
Source: Mol Breed. 2015 Mar 15;35(3):100. doi: 10.1007/s11032-015-0295-4 (PMC4359708; doi:10.1007/s11032-015-0295-4)
Supplement: Supplementary file 1 — Supplementary material 1 (DOCX 127 kb) [file 11032_2015_295_MOESM1_ESM.docx]

**Supplemental Fig. S1 Enzymes and their pathways in sugarcane glycolysis and sucrose accumulation and degradation.**

Phosphomannose isomerase (PMI, EC5.3.1.8); Hexokinase (HXK, E.C. 2.7.1.1); Pyruvate kinase (PK, EC 2.7.1.40); Sucrose phosphate synthase (SPS: E.C. 2.4.1.14); Sucrose synthase (SuSy, E.C. 2.4.1.13); Suc;Sucrose; Fru: Frucrose; Glu: Glucrose; Man-Mannose.
